# The Brand Scandal Spillover Effect at the Country Level: Evidence From Event-Related Potentials

**DOI:** 10.3389/fnins.2019.01426

**Published:** 2020-01-21

**Authors:** Bonai Fan, Chen Li, Jia Jin

**Affiliations:** ^1^School of Public Affairs, Zhejiang University, Hangzhou, China; ^2^Zhejiang Research Base for China’s Non-public Economic Personages, Ningbo University, Ningbo, China; ^3^Academy of Neuroeconomics and Neuromanagement, Ningbo University, Ningbo, China; ^4^School of Business, Ningbo University, Ningbo, China; ^5^China Telecom Corporation Limited, Wuhan, China

**Keywords:** spillover effect, P2, LPP, expectancy violations theory, brand scandal

## Abstract

The spillover effect of brand scandals commonly exists, and this effect will damage the image of the company, industry or even country in which the scandal occurred. Most previous studies on the brand scandal spillover effect have mainly focused on the corporate and industry levels. However, with the development of brand internalization and media technology, the spillover effect at the country level is becoming increasingly common. In the current study, we conducted an event-related potentials study to explore the spillover effect of brand scandals on the country level as well as its underlying neural basis. Specifically, we compared consumers’ attitudes toward countries of origin with different stereotypes during different types of brand scandals. When a competence scandal took place in a competence stereotype country, a larger P2 mean amplitude was elicited compared to a warmth stereotype country. When a morality scandal took place in a warmth stereotype country, a larger LPP mean amplitude was induced compared to a competence stereotype country. We explain the current results based on expectancy violations theory. When competence scandals take place in competence stereotype countries, there will be a greater degree of violation of expectations compared with that in warmth stereotype countries, which leads to a negative evaluation of the country of origin. When morality scandals take place in warmth stereotype countries, people had a stronger negative emotional arousal when morality scandals happened in the warmth stereotype country.

## Introduction

The term spillover effect refers to the phenomenon in which an event influences beliefs regarding attributes that are not directly associated with the event itself (Ahluwalia et al., 2001). Previous studies have documented the spillover effects of brand scandals, either from a partner brand to a host brand or from one brand to a competing brand (Dahlen and Lange, 2006; Votola and Unnava, 2006). Furthermore, some studies have also proposed that the spillover effect can spread to the industry or even to the country level (Roehm and Tybout, 2006; Magnusson et al., 2013). For instance, Magnusson et al. (2013) found that negative events of typical brands will lead to a negative evaluation of the image of the country of origin (Magnusson et al., 2013). Moreover, brand scandal spillover effects at the country level are also found in the real marketplace. For instance, Volkswagen, a world-renowned brand in rigorous and efficient Germany, was dragged into the Dieselgate scandal in 2015, and this scandal not only caused great financial loss and damaged the reputation of Volkswagen but also threatened the “Made in Germany” image (Chambers, 2015).

Recently, with the sustained development of brand internalization and new media technologies, spillover effects at the country level have become more common and more serious than ever before (Magnusson et al., 2013). However, most previous studies have mainly focused on the spillover effect at the brand level or the industry level (Votola and Unnava, 2006; Lei et al., 2008), with little concern for the brand scandal spillover effect at the country level. Thus, more related research is needed to better understand the underlying basis of the spillover effect at the country level to deal with it. Therefore, in the current study, we intend to study the spillover effect of brand scandals at the country level.

According to previous studies, spillover effects are not a fixed phenomenon; rather, they can be affected by the scandal type. For example, Votola and Unnava (2006) discussed how types of brand negative effects (e.g., the competence type and the moral type)influenced the spillover effect of negative information on host brands in brand alliances. They found that negative information regarding competence is more harmful to the host brand than morality when the partner is a company, and vice versa when the partner is a spokes person (Votola and Unnava, 2006). It seems that competence and morality scandals have different spillover effects based on the different types of partner brands at the brand level. Thus, when we study the spillover effect at the country level, it is natural to ask whether these two types of scandals will have different spillover effects based on the different types of countries of origin.

On the other hand, previous studies have not only supported the notion that there exist different types of countries of origin but also suggested that different types of countries can influence consumers’ impressions of products (Kramer et al., 2008; Magnusson et al., 2013). For example, Kramer et al. (2008) divided national stereotypes into perceived warmth or competence and explored the interaction effect between product type and national stereotype. They found that products from countries that were stereotyped as competent are perceived as being more utilitarian than hedonic, while products are perceived as being more hedonic than utilitarian when they are from a country with a warmth stereotype (Kramer et al., 2008).

These studies suggested that when evaluating products, country of origin stereotype image can set up expectations about product features in consumers’ mind. Burgoon (1978) proposed Expectancy Violations Theory in 1987, which mentioned that expectations are generally based on the standards of social norms and known features (Burgoon, 1978), and consumers may often automatically draw on stereotypes when making decisions (Devine, 1989). Thus, consumers will rely on national stereotypes when evaluating country image. In the current study, we intend to examine the spillover effect on competence stereotype countries and warmth stereotype countries after brand scandals involving either morality or competence.

Previous studies on brand scandal spillover effects have mainly employed behavioral measures (Ahluwalia et al., 2000; Votola and Unnava, 2006). With the development of neuroscience technology, neuroscientific tools have recently been used in the marketing area since they can provide a more direct approach for measuring consumers’ cognitive processes (Shiv and Yoon, 2012; Yoon et al., 2012). For instance, Jin et al. (2015) used event-related potentials (ERPs) to study consumers’ evaluation of brand strategy. They found that the amplitude of the negative N400 component varied according to consumers’ perceived fitness between brand name and product name (Jin et al., 2015). Min et al. (2014) used ERPs to investigate the association between the country of origin and consumers’ evaluation of a product design, finding that the N90 and P220 components are involved in original design evaluation, whereas the later P500 can reflect the cognitive assessment of the country of origin (Min et al., 2014). Therefore, in the current study, we also intend to use ERPs to explore the cognitive processes of the brand scandal spillover effect at the county level to better understand its underlying mechanism.

In previous consumer neuroscience studies, P2 and LPP are two ERP components that have always been used to examine consumers’ evaluations of marketing stimuli (Carretié et al., 2001; Chen et al., 2010; Bublatzky and Schupp, 2012). P2 was found to peak approximately 200 ms after the onset of stimuli and was mainly distributed in the frontal area (Crowley and Colrain, 2004; Polezzi et al., 2008). P2 is an attention-related component, reflecting early rapid automatic activity (Crowley and Colrain, 2004; Polezzi et al., 2008), and an enhanced P2 reflects the engagement of attentional resources (Carretié et al., 2001; Chen et al., 2010; Bublatzky and Schupp, 2012). Furthermore, it has been reported that negative stimuli can engage more attentional resources compared to positive stimuli, resulting in negative stimuli that induce larger P2 amplitudes than positive stimuli (Carretié et al., 2001; Huang and Luo, 2006; Thomas et al., 2007). In the consumer neuroscience domain, researchers use this feature of P2 to reveal consumers’ evaluation of marketing-related stimuli. For example, Jin et al. (2017) found that larger P2 amplitudes were elicited by negative framing information compared to positive framing information when describing a product in an online shopping website even when the positive and negative frames carried the same meaning. This result indicates that consumers have a more negative assessment of negative framing information at the early stage of rapid automatic processing (Jin et al., 2017). Therefore, the larger amplitude of P2 may reflect a more negative evaluation of a country at the early stage in the current study.

Another relevant ERP component is LPP, which typically peaks approximately 600 ms after stimulus onset in the central-parietal region (Cuthbert et al., 2000; Schupp et al., 2006; Herring et al., 2011). Previous studies have reported that the amplitude of LPP is an indicator of emotional arousal, and high arousal stimuli can cause larger LPP amplitudes than low arousal stimuli (Cuthbert et al., 2000; Schupp et al., 2000). In particular, a significant pleasant or unpleasant stimulus can elicit a larger amplitude of LPP compared to neutral visual stimuli (Olofsson et al., 2008). Moreover, Ma et al. (2010) found that prior high hazard words elicited a larger LPP amplitude than prior low hazard words, indicating that prior high hazard words have a higher strength of negative emotion, resulting in a larger LPP amplitude (Ma et al., 2010). Therefore, as all the stimuli in the current study are negative brand scandal events, a larger amplitude of LPP reflects a higher emotional arousal induced by more negative stimuli.

As discussed above, we hypothesize that a brand scandal will spill over to the country level and that the national stereotype and the scandal type will influence the degree of the spillover effect. Furthermore, this effect can be discovered not only by behavioral results but also by the deflection of P2 and LPP amplitudes. Specifically, P2 can reflect an early evaluation of the country image after scandal events. Based on expectation violation theory (Burgoon, 1978), we considered that consumers will rely on national stereotypes when evaluating country image. If consumers have a more negative evaluation toward competence stereotype country than warmth stereotype country when competence scandal happened, then a larger P2 will be elicited. And if consumers have a more negative evaluation toward warmth stereotype country than competence stereotype country when morality scandal happened, it will also elicit a larger P2. For LPP, it can reflect the emotional arousal of the stimuli (Cuthbert et al., 2000; Schupp et al., 2000). If consumers have a more enhanced emotional arousal when competence scandal happened in competence stereotype country than in a warmth country, then a larger LPP will be elicited. And if consumers have a more intense emotional arousal when morality scandals take place in warmth stereotype countries than in competence stereotype country, then a larger LPP will be elicited.

## Materials and Methods

### Participants

Fifteen (eight males and seven females) healthy right-handed graduate or undergraduate students from Ningbo University participated in this study. All participants were 18–24 (*M* = 19.928, *SD* = 1.774) years of age, had normal or corrected-to-normal vision and had no history of neurological problems. All participants provided written informed consent before the experiment started, and they each received 40 yuan after the completion of the experiment as reward. This study was also approved by the Ethical Committee of the Academy of Neuroeconomics and Neuromanagement at Ningbo University.

### Stimulus Design

The stimulus contained two types of countries (a competence stereotype country and a warmth stereotype country) with eight brand scandal events (four morality scandal events and four competence scandal events). Each stimulus was repeated 10 times, thus, 160 stimuli were contained in the entire experiment. In the experiment, we referred to the competence stereotype country as country A and the warmth stereotype country as country B. To give the participants an association with the two country types and their names, a cover story describing the two distinct dimensions of national stereotypes was provided before the experiment. In the cover story, we depicted country A as possessing the characteristics of competence, capability and efficiency. The description of country B was focused on creating a friendly, warm, kind image. To ensure that the participants had read the material carefully and could distinguish between the stereotypes, we interviewed each participant to verify that they had the desired representations of the two countries in mind before the formal experiment started, and we also ask the participants to complete a questionnaire scoring the competence and warmth stereotype countries using a seven-point Likert scale (7 = “extremely good” to 1 = “extremely bad”). The result showed that the impressions of the two countries were not significantly different [*t*(14) = −1.148, *p* > 0.1].

According to previous studies, morality scandals are scandals caused by social and value-related reasons, while competence scandals are primarily caused by product defects (Wojciszke et al., 1993; Votola and Unnava, 2006). Therefore, in the current experiment, the four morality scandal events were a sewage spill, design plagiarism, labor abuse and the poaching ofen dangered species, while the competence scandal events were unsafe formaldehyde levels, poor colorfastness, a lowered pH level, and fabric impurity. We interviewed the experimental participants in advance to ensure that all participants agreed that each of the events noted above would represent competence or morality brand scandals.

All 160 stimuli were randomly separated into four blocks, with each block consisting of 40 trials. In each block, all trials were presented randomly. The size of each picture was 270 × 360 pixels, and they were shown on a gray background.

### Experimental Procedure

The participants were asked to sit in a dim, sound-attenuated, electrically shielded room. The visual stimuli were presented centrally on a computer-controlled monitor (1280 × 1024 pixels, 60 Hz) at a distance of 100 cm from the participant and with a visual angle of 2.588°. The participants were provided with a keypad to score the country image by pressing a key (key 1 for decrease, key 3 for increase and key 2 for confirmation). Before the formal experiment started, all participants were given a brief introduction about the experimental process. After they fully understood the process, the experiment started.

A fixation cross appeared at the beginning of each trial for 600–800 ms on a blank screen, indicating the start of a trial. Next, an image with a country name appeared for 1500 ms, followed by a blank screen for 400–600 ms. The content of the brand scandal was subsequently shown for 1500 ms. Then, the sentence “Please rate the image of the country” appeared, and the participants had to rate the country image from 1 “very bad” to 7 “very good” after the scandal happened by pressing a button. The stimuli disappeared immediately after the button was pressed. The participants were asked to minimize eye and muscle movement during the experiment. The E-Prime 2.0 software package (Psychology Software Tools, Pittsburgh, PA, United States) was used for stimulus presentation, triggers and response recording. To become familiar with the experimental procedure, all participants had eight practice trials before the experiment formally began.

### EEG Recordings

Electroencephalogram (EEG) signals were recorded using a cap containing 64 Ag/AgCl electrodes and a Neuroscan Synamp2 Amplifier (Curry 7, Neurosoft Labs, Inc.) with a sample rate of 1000 Hz and a bandpass filter from 0.05 to 70 Hz. A cephalic (forehead) location was used as a ground. There were two mastoid electrodes, and the left mastoid was selected as an online reference. Horizontal and vertical electrooculograms (EOG) were monitored with two pairs of electrodes. The vertical EOG was recorded 10 cm supra- and infra-orbitally at the left eye, and the horizontal EOG was recorded 10 cm to the left and right of the lateral canthi of both eyes. The experiment started only when the electrode impedances were kept under 5 kΩ.

### Data Analysis

To analyze the behavioral data, a repeated-measures ANOVA was used. We compared therating scores in four conditions with a 2(brand scandal: morality scandal and competence scandal) × 2(national stereotype: warmth and competence) design.

The EEG recordings were analyzed using Curry 7 (Neurosoft Labs, Inc.). The data were rereferenced to the algebraically computed average of the left and right mastoids for further analysis. EOG artifacts were corrected during preprocessing using the method proposed by Semlitsch et al. (1986). The data were then digitally filtered with a low-pass filter at 30 Hz (24 dB/octave) and segmented for the epoch from 200 ms before the onset of the target appearing on the video monitor to 800 ms after onset, with the first 200 ms pretarget used as the baseline. Trials containing amplifier clipping, bursts of electromyography activity, or peak-to-peak deflection exceeding ± 100 V were excluded. The EEG recordings from each recording site for every participant were averaged separately for the four conditions, and numbers of ERP trials retained for analysis for every participants were showed as follow: (1) 27–40 trials (*M* = 36.07, *SD* = 4.20) for the warmth stereotype country with a competence scandal; (2) 25–40 trials (*M* = 36.33, *SD* = 4.67) the warmth stereotype country with a morality scandal; (3) 27–40 trials (*M* = 36.67, *SD* = 4.29) the competence stereotype country with a competence scandal; (4) 22–40 trials (*M* = 35.80, *SD* = 5.73) the competence stereotype country with a morality scandal.

Based on the visual observation and guidelines provided by Picton et al. (2000), we analyzed two ERP components: P2 and LPP. We chose a time window range of 240–320 ms and nine electrodes (F1, Fz, F2, FC1, FCz, FC2, C1, Cz, and C2) in the frontal-central areas for the P2 mean amplitude statistical analysis. To investigate the effect across conditions, a 2(brand scandal: morality scandal and competence scandal) × 2(national stereotype: warmth and competence) × 9 (electrodes: F1/z/2, FC1/z/2, and C1/z/2) repeated-measures ANOVA was performed for the P2 amplitude. Similarly, time windows of 580–660 ms and six electrodes (CP1, CPz, CP2, P1, Pz, and P2) in the parietal–occipital areas were selected for the LPP analysis, and a 2(brand scandal: morality scandal and competence scandal) × 2(national stereotype: warmth and competence) × 6 (electrode: CP1/z/2 and P1/z/2) repeated-measures ANOVA was also conducted for the LPP amplitudes. A simple effects analysis was conducted when the interaction effect was significant. The Greenhouse–Geisser correction (Greenhouse and Geisser, 1959) was applied in all statistical analyses when necessary (uncorrected dfs are reported with ε and the corrected *p*-values). And *p*-value less than 0.05 represents a significant effect, between 0.05 and 0.10 represents a marginal significant effect (Aaker and Lee, 2001).

## Results

### Behavioral Results

**FIGURE 1:**
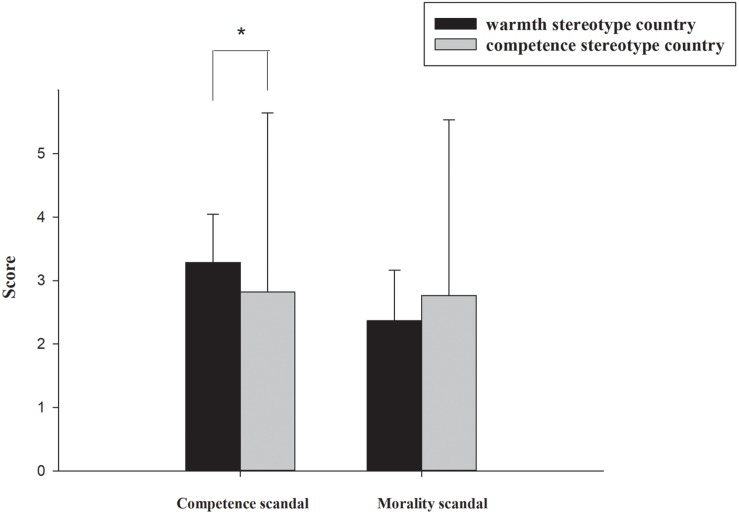
Behavioral results of the country impression evaluation: the country impression scores of the warmth stereotype country and the competence stereotype country under competence brand scandals and morality brand scandals. ^∗^*p* < 0.05; ^∗∗^*p* < 0.001.

The behavioral results are shown in Figure 1. A 2 (brand scandal: morality scandal vs. competence scandal) × 2 (national stereotype: warmth vs. competence) repeated-measures ANOVA of the scores was carried out, and the results revealed that there was no significant main effect for country of origin [*F*(1,14) < 1, *p* > 0.1]. However, the main effect for brand scandals was significant [*F*(1,14) = 7.647, *p* = 0.015], and the mean score for morality scandals conditon (*M* = 2.567, *SD* = 0.195) was lower than that for competence scandals (*M* = 3.054, *SD* = 0.119). The interaction effect between brand scandal and national stereotype was significant [*F*(1,14) = 8.405, *p* = 0.012]. Furthermore, we conducted a simple effects analysis to determine the effects of different countries with fixed brand scandal content. In the competence scandal condition, there was a main effect of country of origin [*F*(1,14) = 4.508, *p* = 0.052]. The mean scores for the competence stereotype country (*M* = 2.822, *SD* = 0.453) were lower than those for the warmth stereotype country (*M* = 3.287, *SD* = 0.759). However, in the morality scandal condition, there was no significant main effect of national stereotype [*F*(1,14) = 3.500, *p* = 0.082].

### P2

**FIGURE 2:**
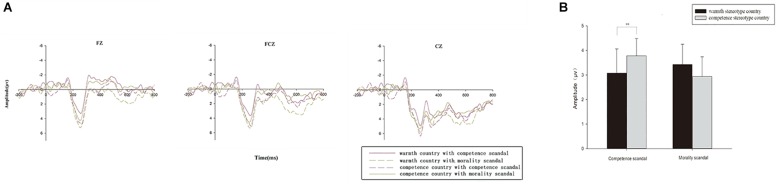
**(A)** Grand average P2 waveforms in the frontal areas in representative electrodes from channels Fz, FCz, and Cz, which stand for the selected nine electrodes as a comparison for the following four conditions: (1)the warmth stereotype country with a competence scandal; (2) the warmth stereotype country with a morality scandal; (3) the competence stereotype country with a competence scandal; (4) the competence stereotype country with a morality scandal. **(B)** Grand average bar graph for P200: the grand average amplitude of P2 of the warmth stereotype country and the competence stereotype country under competence brand scandals and morality brand scandals. ^∗^*p* < 0.05; ^∗∗^*p* < 0.001.

As shown in Figure 2, the three-way 2 (brand scandal) × 2 (national stereotype) × 9 (electrodes) repeated-measures ANOVA for the P2 amplitude produced no significant main effect for either the brand scandal type [*F*(1,14) = < 1, *p* > 0.1, $\eta_{\text{p}}^{2}$ = 0.027] or national stereotype [*F*(1,14) = < 1, *p* > 0.1, $\eta_{\text{p}}^{2}$ = 0.085]. However, a significant interaction effect between brand scandal and national stereotype was observed [*F*(1,14) = 5.513, *p* = 0.034, $\eta_{\text{p}}^{2}$ = 0.283].

To examine this interaction, a simple effects analysis was conducted. When the brand scandal was related to competence, the effect of national stereotype was significant [*F*(1,14) = 6.076, *p* = 0.027, $\eta_{\text{p}}^{2}$ = 0.303]. The results suggested that the scandals of competence stereotype country (*M* = 3.789 μV, *SE* = 0.707) elicited a larger P2 mean amplitude (positive polarity: a larger voltage value means a larger amplitude) compared to the morality stereotype country (*M* = 3.087 μV, *SE* = 0.981). However, for the morality scandal condition, the mean amplitude of P2 between the warmth stereotype country and the competence stereotype country was not significantly different [*F*(1,14) = 2.197, *p* > 0.1, ${\text{η}}_{\text{p}}^{2}$ = 0.136].

And we also conducted simple effect test on the P2 amplitudes between competence and warmth stereotype country conditions separately when competence and morality brand scandal occurred. The results showed that, for P2, when the country stereotype was related to warmth, the effect of brand scandal was significant [*F*(1,14) = 7.779, *p* = 0.014, $\eta_{\text{p}}^{2}$ = 0.357]. The results suggested that the morality scandal (*M* = 3.953 μV, *SE* = 1.070) elicited a larger P2 mean amplitude (positive polarity: a larger voltage value means a larger amplitude) compared to the competence scandal (*M* = 2.323 μV, *SE* = 1.214). However, for the competence stereotype country condition, the mean amplitude of P2 between the morality scandal and competence scandal was not significantly different [*F*(1,14) = 1.984, *p* > 0.1, $\eta_{\text{p}}^{2}$ = 0.124].

### LPP

**FIGURE 3:**
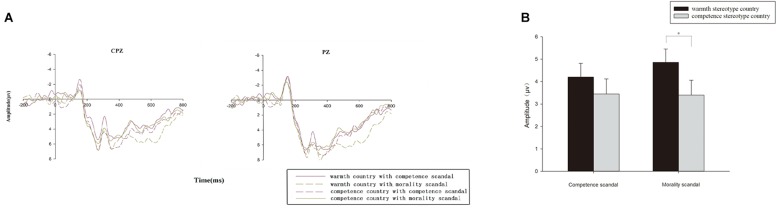
**(A)** Grand average LPP waveforms in the central-parietal areas in representative electrodes from channels CPz and Pz, which stand for the selected six electrodes as a comparison for the following four conditions: (1) the warmth stereotype country with a competence scandal; (2) the warmth stereotype country with a morality scandal; (3) the competence stereotype country with a competence scandal; (4) the competence stereotype country with a morality scandal. **(B)** Grand average bar graph for LPP: the grand average amplitude of LPP of the warmth stereotype country and the competence stereotype country under competence brand scandals and morality brand scandals. ^∗^*p* < 0.05; ^∗∗^*p* < 0.001.

For the parietal–occipital component LPP, a three-way 2 (brand scandal) × 2 (national stereotype) × 6 (electrodes) repeated-measures ANOVA in the time window from 580 to 660 ms identified no significant main effect for either the brand scandal type [*F*(1,14) = 2.204, *p* > 0.1, $\eta_{p}^{2}$ = 0.136] or national stereotype [*F*(1,14) = 2.204, *p* > 0.1, $\eta_{\text{p}}^{2}$ = 0.136]. Moreover, the interaction effect between brand scandal and national stereotype was marginally significant [*F*(1,14) = 4.502, *p* = 0.052, $\eta_{\text{p}}^{2}$ = 0.243]. A simple effects analysis revealed that in the competence scandal condition, the mean voltage of the LPP amplitude (positive polarity: a larger voltage value means a larger amplitude) was not significant [*F*(1,14) = 0.055, *p* > 0.1, $\eta_{\text{p}}^{2}$ = 0.004] between the warmth stereotype country and the competence stereotype country. However, for the morality scandal condition, there was a significant effect of national stereotype [*F*(1, 14) = 4.964, *p* = 0.043, $\eta_{\text{p}}^{2}$ = 0.262], with the warmth stereotype country (*M* = 4.860 μV, *SE* = 0.594) showing a larger LPP mean amplitude compared to the competence stereotype country (*M* = 3.401 μV, *SE* = 0.663), as shown in Figure 3.

When the country stereotype was related to warmth, the effect of brand scandal was significant [*F*(1,14) = 8.643, *p* = 0.011, $\eta_{\text{p}}^{2}$ = 0.382]. The results suggested that the morality scandal (*M* = 5.637 μV, *SE* = 0.729) elicited a larger LPP mean amplitude (positive polarity: a larger voltage value means a larger amplitude) compared to the competence scandal (*M* = 3.402 μV, *SE* = 0.659). However, for the competence stereotype country condition, the mean amplitude of LPP between the morality scandal and competence scandal was not significantly different [*F*(1,14) = 0.179, *p* > 0.1, $\eta_{\text{p}}^{2}$ = 0.013].

## Discussion

Using ERPs and a lab experiment, the present study explored the neural evidence of the spillover effect of brand scandals at the country level. Specifically, we discussed the influence on country image when different types of brand scandals took place in different countries of origin. The behavioral results showed that the overall scores for morality scandals were lower than those for competence scandals, indicating that morality scandals induce a more negative evaluation of the country of origin than do competence scandals. This finding suggests that it is easier for a morality scandal to spill over to the country level. This is consistent with previous studies, which showed that people are more likely to show dissatisfaction with morality scandals than with competence scandals (Kanouse and Hanson, 1987; Kervyn et al., 2014). Further analysis showed that when a competence scandal occurred, the participants’ rating of the competence stereotype country was significantly lower than that of the warmth stereotype country. However, when a morality scandal occurred, the differences between the two types of countries were not significant. These results suggest that it is easier for competence scandals to spill over to affect the image of a competence stereotype country compared to a warmth stereotype country. The ERP results showed that when a competence scandal took place in a competence stereotype country, a larger P2 mean amplitude would be elicited compared to a warmth stereotype country. However, for morality scandals, no significant distinction between the competence stereotype country and the warmth stereotype country was found. As mentioned in the introduction, a larger amplitude of P2 reflects a more negative attitude toward a stimulus. Thus, these results reflect that, compared with a warmth stereotype country, consumers’ evaluation of a competence stereotype country is more negative when a competence brand scandal occurs. This result is consistent with the behavioral results, which reflect that competence scandals spill over to the competence stereotype country more easily than to the warmth stereotype country. In addition, for morality scandals, the mean amplitude of LPP was significantly larger (positive polarity) for the warmth stereotype country than for the competence stereotype country. However, in the context of competence brand scandals, there is no significant difference between a competence stereotype country and a warmth stereotype country. Previous studies have shown that an increased LPP amplitude could be interpreted as an index of emotional arousal at the late cognition processing stage (Cuthbert et al., 2000; Schupp et al., 2000). Because brand scandals are all associated with negative emotion, a larger LPP amplitude indicates a more intense negative emotional arousal. The current LPP results show that in the context of a morality scandal, more negative emotion is evoked when the country of origin is a warmth stereotype country than when it is a competence stereotype country. Meanwhile, in the context of a competence brand scandal, there is no difference in the negative emotion evoked by a competence stereotype country and by a warmth stereotype country.

We consider that expectancy violations theory (Burgoon, 1978; Jussim et al., 1987) can explain the spillover effect. Acts that violate expectations will result in negative evaluations and affective response (Coles, 1989). Consumers also have expectations for the national image of a country, and these expectations are dependent on the existing national stereotype (Maheswaran, 1994). Previous studies have indicated that when brand scandals contradict national stereotypes, they enhance peoples’ concerns and thus impact the evaluation of national image (Bond et al., 1992).

In the current study, since competence stereotype countries are often considered to be competent, capable and efficient (Fiske et al., 2002), when a competence scandal occurred, it contradicted national stereotypes, resulting in a violation of consumers’ expectations. As a result, it was easier for the competence scandal to spill over to the competence stereotype country compared with the warmth stereotype country. However, for the warmth country, competence scandals had no relationship with the country’s national stereotype, which is often considered to be sincere and friendly (Fiske et al., 2002). As shown in Figure 2A, the P2 amplitudes in the morality scandal conditions are relatively larger in both country types. This result means that when a morality scandal occurred, both countries violate consumers’ expectations. This is consistent with a previous study that demonstrated that every person in society is expected to observe certain moral standards (Kanouse and Hanson, 1987). Therefore, morality scandals will affect the impression of both types of countries.

Furthermore, as shown in Figure 3A, we found that morality scandals in the warmth stereotype country had the largest LPP amplitude. This finding indicates that people had a stronger negative emotional arousal when morality scandals happened in the warmth stereotype country. Since warmth stereotype countries are often considered to be sincere and friendly, when a morality brand scandal happens, people will feel hurt and disappointed in the country (Wojciszke et al., 1993), leading to stronger negative emotional arousal. This is similar to previous studies on gender stereotypes in politics, in which women are assumed to be honest and rigid in fulfilling moral standards (Kahn, 1992) and, therefore, female politicians involved in corruption and sex scandals are treated more harshly than male politicians involved in such scandals (Żemojtel-Piotrowska et al., 2017). However, regarding competence scandals, as such a scandal is more likely to be forgiven (Kanouse and Hanson, 1987), it will induce relatively weak negative emotional arousal when it happens. This is consistent with the study by Kervyn et al. (2014), who used the BP oil spill as an example. They found that if there is a problem with ability, the brand’s reputation will be repaired more easily and lead to a more tolerant judgment from consumers (Kervyn et al., 2014).

## Conclusion

In summary, this study investigated the underlying neural mechanisms of the brand scandal spillover effect and found differences in country-level spillover effects between different brand scandal types. The behavioral results showed that participants had a worse impression when a competence scandal happened in a competence stereotype country than when it happened in a warmth stereotype country. The ERP results indicated that when a competence scandal occurred, a competence stereotype country could induce a more negative evaluation than could a warmth stereotype country (larger P2 amplitude). When a morality scandal occurred, both countries were subject to similarly negative evaluations. Moreover, compared with the competence stereotype country, the warmth stereotype country induced higher emotional arousal (larger LPP amplitude) in the context of a morality scandal. We explain the spillover effect based on expectancy violations theory and identify the existence of the spillover effect at the national level. Furthermore, the current results also highlight the differences in early automatic evaluation and later emotional arousal when consumers face different types of scandals in different countries.

## Data Availability Statement

The raw data supporting the conclusions of this manuscript will be made available by the authors, without undue reservation, to any qualified researcher.

## Ethics Statement

The studies involving human participants were reviewed and approved by the Ethics Committee of the Ningbo University. All subjects gave written informed consent in accordance with the Declaration of Helsinki.

## Author Contributions

CL and BF conceived and designed the study. CL collected and analyzed the data. CL and JJ interpreted the data and drafted the manuscript. BF, JJ, and CL reviewed and edited the manuscript. BF administered the project.

## Conflict of Interest

CL was employed by China Telecom Corporation Ltd. The remaining authors declare that the research was conducted in the absence of any commercial or financial relationships that could be construed as a potential conflict of interest.
